# Oxygen Incorporation as a Route to Nondegenerate Zinc Nitride Semiconductor Thin Films

**DOI:** 10.1021/acsami.4c16921

**Published:** 2025-01-28

**Authors:** Elise Sirotti, Bianca Scaparra, Stefan Böhm, Florian Pantle, Laura I. Wagner, Felix Rauh, Frans Munnik, Chang-Ming Jiang, Matthias Kuhl, Kai Müller, Johanna Eichhorn, Verena Streibel, Ian D. Sharp

**Affiliations:** † Walter Schottky Institute, Technical University of Munich, Garching 85748, Germany; ‡ Physics Department, TUM School of Natural Sciences, Technical University of Munich, Garching 85748, Germany; § TUM School of Computation, Information and Technology, and MCQST, Technical University of Munich, Garching 85748, Germany; ∥ Helmholtz-Zentrum Dresden-Rossendorf, Dresden 01328, Germany

**Keywords:** zinc nitride, defect properties, molecular beam epitaxy, electronic properties, Hall effect

## Abstract

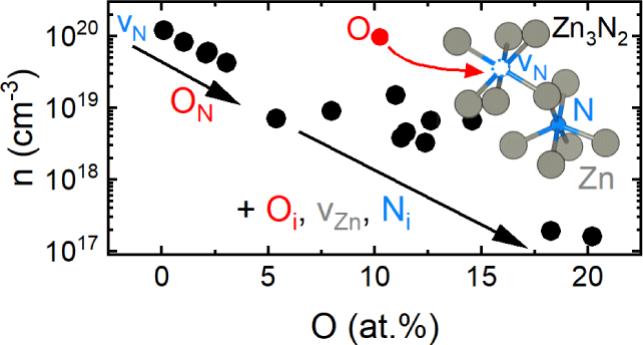

Zinc nitride (Zn_3_N_2_) comprises earth-abundant elements, possesses a small direct bandgap, and is characterized by high electron mobility. While these characteristics make the material a promising compound semiconductor for various optoelectronic applications, including photovoltaics and thin-film transistors, it commonly exhibits unintentional degenerate n-type conductivity. This degenerate character has significantly impeded the development of Zn_3_N_2_ for technological applications and is commonly assumed to arise from incorporation of oxygen impurities. However, consistent understanding and control of the role of native and impurity defects on the optoelectronic properties of this otherwise promising semiconductor have not yet emerged. Here, we systematically synthesize epitaxial Zn_3_N_2_ thin films with controlled oxygen impurity concentrations of up to 20 at % by plasma-assisted molecular beam epitaxy (PA-MBE). Contrary to expectations, we find that oxygen does not lead to degenerate conductivity but instead serves as a compensating defect, the control of which can be used to achieve nondegenerate semiconducting thin films with free electron concentrations in the range of 10^17^ cm^–3^, while retaining high mobilities in excess of 200 cm^2^ V^–1^ s^–1^. This understanding of the beneficial role of oxygen thus provides a route to controllably synthesize nondegenerate O-doped Zn_3_N_2_ for optoelectronic applications.

## 1 Introduction

The development of electronic materials that feature efficient optical absorption and electronic transport properties, while also comprising earth-abundant elements and compatibility with low-temperature production, is important for ensuring a sustainable future. Yet, many of the highest-performance compound semiconductors in use today are based on scarce, expensive, and sometimes toxic elements.^1^ To address this challenge, a broad range of oxides have been extensively investigated and offer routes to scalable processing. However, most of these materials possess wide bandgaps and suffer from sluggish charge transport and short photocarrier lifetimes. By comparison, metal nitrides tend to have narrower bandgaps due to the energetically higher-lying N 2p orbitals that comprise their valence bands, and larger carrier mobilities due to their more covalent bonding and weaker electron–phonon interaction strengths.^2,3^ Despite these advantages, nitride-based semiconductors remain difficult to reliably synthesize due to the needed activation of strong dinitrogen bonds and the propensity for oxygen incorporation during synthesis.^2,4^ While such challenges have been overcome for the technologically important class of III–N compounds, the range of functional nitride semiconductors with tunable electronic properties remains limited.

Within this class of materials, Zn_3_N_2_ is an extensively studied but poorly understood compound semiconductor. While its theoretically predicted direct bandgap of 1.0 eV and reported mobilities of up to 395 cm^2^V^–1^s^–1^ are promising for application in thin-film solar cells and transistors,^5−7^ Zn_3_N_2_ is often reported to be a degenerate semiconductor, limiting its practical functionality. Moreover, the reported properties of Zn_3_N_2_ thin films vary greatly depending on the synthesis route, with previous studies including thin film growth by molecular beam epitaxy (MBE),^6,8,9^ sputter deposition,^10−13^ and chemical reaction methods.^14−16^ The experimentally observed bandgaps vary widely between 1.0 and 3.2 eV. While 1.0 eV is similar to the theoretically predicted value,^14,17^ 3.2 eV is close to the bandgap of ZnO (3.4 eV).^18^ Thus, the larger reported values likely reflect the rapid oxidation of Zn_3_N_2_ in air,^19,20^ rendering the smaller values, near 1.0 eV, to be more reflective of the pristine, nonoxidized form of Zn_3_N_2_.

Despite the desirable narrow bandgap and high mobilities of Zn_3_N_2_, the main impediment to its use in optoelectronic applications is the lack of reliable approaches to control its conductivity. In particular, reported charge carrier concentrations of Zn_3_N_2_ thin films range from 10^17^ to 10^20^ cm^–3^,^6,11^ with the majority of studies observing unintentional degenerate n-type character. In addition, only a few studies exist on epitaxially grown Zn_3_N_2_ layers, which are extremely valuable for elucidation and control of fundamental material properties, especially with respect to charge transport characteristics. To the best of our knowledge, three groups have used MBE to grow epitaxial Zn_3_N_2_ thin films,^6,8,9^ while one group has done so using radio frequency (rf) magnetron sputtering.^11^ In all cases, electrical measurements of these epitaxially grown samples reveal degenerate behavior, with charge carrier concentrations ranging from 10^18^ to 10^20^ cm^–3^.^6,8,9,11^ Likewise, polycrystalline thin films grown using different approaches also tend to exhibit degenerate character, though these reports have yielded a broader range of charge carrier concentrations that extend down to 10^17^ cm^–3^.^11,12,20,21^ Although systematic studies of impurities and native defects in Zn_3_N_2_ remain lacking, the high electron concentration is commonly attributed to oxygen impurities, a donor element that tends to bind with zinc.^22^ For example, Cao et al. and Suda et al. observed an increase in the charge carrier concentration from 3 × 10^19^ cm^–3^ to 2 × 10^20^ cm^–3^ with increasing oxygen content.^17,23^ Likewise, Oshima et al.^9^ assigned the source of the degenerate conductivities of their MBE-grown Zn_3_N_2_ films to oxygen impurities. In other studies, Wang et al.^12^ and Li et al.^24^ reported polycrystalline Zn_3_N_2_ thin films sputtered at room temperature with bandgaps of 1.0 eV and charge carrier concentrations in the range of 10^17^ cm^–3^, resulting in nondegenerate semiconducting character. The authors hypothesized that this low growth temperature reduced the probability for formation of unintentional donor defects, such as substitutional oxygen on nitrogen sites (O_N_) or hydrogen interstitials (H_i_), thus enabling comparatively low electron concentrations. However, precise composition analyses of these different Zn_3_N_2_ layers have not been systematically reported, complicating comparisons between different published studies. Notably, only reactive sputtering has resulted in nondegenerate films, despite this method typically yielding larger impurity (e.g., oxygen) concentrations than MBE. The above discussion highlights the critical influence of oxygen impurities on defining the structural, electrical, and optical properties of Zn_3_N_2_, as well as the persistent uncertainties regarding strategies to tune its conductivity.

In the present work, we report a systematic study of the impact of oxygen on the optical and electronic properties of this material. By introducing increasing amounts of oxygen during the growth of high-quality epitaxial oxygen-doped Zn_3_N_2_ (O:Zn_3_N_2_) on *a*-plane sapphire, we show that the free charge carrier concentration can be tuned from 10^20^ cm^–3^, where the material exhibits degenerate character and metallic conductivity, down to 10^17^ cm^–3^, at which point the material is nondegenerate and characterized by semiconducting behavior. Importantly, this trend of decreasing conductivity with increasing oxygen content is opposite to most reports in the literature, where oxygen is commonly assumed to be an n-type dopant.^17,23^ We resolve this apparent paradox by also considering the critical influence of nitrogen vacancies (v_N_), which are important native defects that also act as donors but are significantly more challenging to investigate experimentally. Our results suggest that v_N_ are important donor defects in Zn_3_N_2_ that contribute to its degenerate character, but that they can be partially passivated by O_N_. Thus, while O_N_ is a donor impurity in Zn_3_N_2_, its key role in passivating v_N_ contributes to decreasing the free carrier concentration and enable nondegenerate conductivity, most likely through the formation of additional compensating defects, including interstitial oxygen at structural vacancy sites (O_i_). As such, this work presents a new strategy for precisely controlling the electrical properties of Zn_3_N_2_, thereby providing a route toward functional films offering highly desirable characteristics for optoelectronics and energy conversion applications.

## 2 Results

### 2.1 Structural Properties and Compositional Analysis

Zn_3_N_2_ thin films with variable oxygen content were synthesized by plasma-assisted molecular beam epitaxy (PA-MBE) on *a*-plane sapphire (Al_2_O_3_) at a fixed substrate temperature of 150 °C. A total of 19 films were deposited with variable oxygen concentrations but otherwise fixed growth parameters, as described in Section 5. The thin films are labeled according to their O content, as determined by energy dispersive X-ray spectroscopy (EDX) using a detector mounted on a scanning electron microscope (SEM), following quantitative calibration by elastic recoil detection analysis (ERDA, see Section 5 for details). In addition to the intentionally O-doped films, pure Zn_3_N_2_ reference samples were grown and contain oxygen concentrations of approximately 0.6 at %. These unintentionally doped films are denoted as <1 at % O samples.

**Figure 1:**
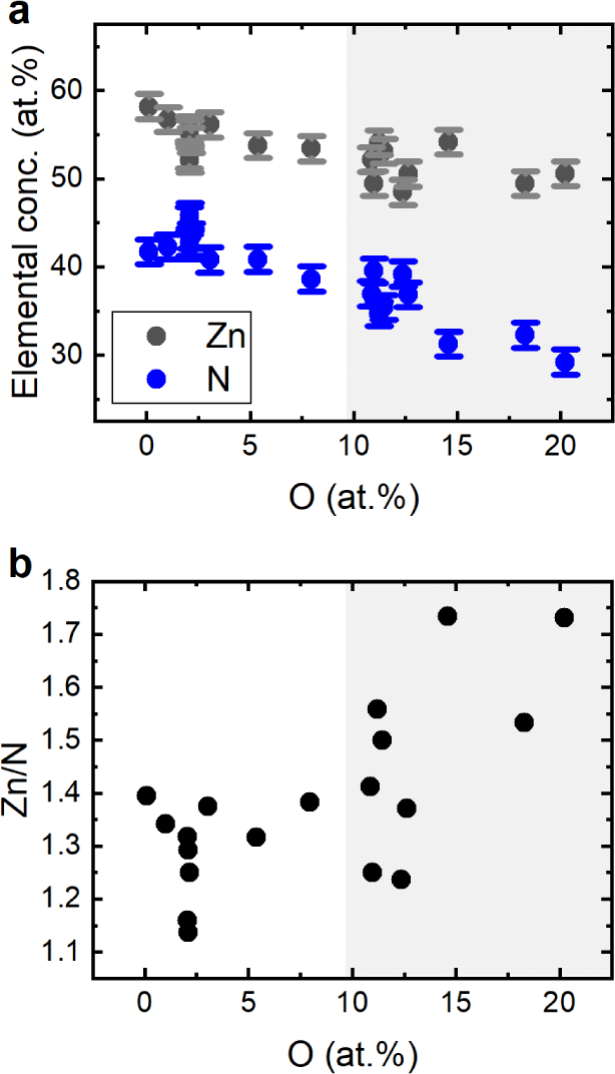
(a) Chemical characterization of O:Zn_3_N_2_ as a function of O content determined by EDX and calibrated based on ERDA results of selected samples, presented as the Zn concentration (gray) and N concentration (blue) as a function of O concentration. (b) Zn/N ratio as a function of the O content determined from the values presented in (a). The gray background is a guide to the eye to emphasize the different regions discussed in the text. Error bars were estimated from the standard deviation obtained from analysis of several samples possessing nearly identical oxygen contents of approximately 2.1 at %.

Figure 1a shows the N and Zn contents as a function of the O concentration within the O:Zn_3_N_2_ thin films. Here, we observe a decrease of the N content from 45 at % to 29 at % with increasing O content, with the linear dependence described by a slope of −0.7 at % N per at % O. While the Zn content shows a similar decreasing trend with increasing O content, it exhibits a significantly weaker dependence, declining from a maximum value of 58 at % at low O content to 49 at % at high O content. This weaker dependence is especially apparent for O concentrations >4 at %, where the fitted slope yields a value of −0.3 at % Zn per at % O. The decreased Zn content corresponds to an increase of total anion (O + N) content from 42 at % to 51 at %. While the above dependencies of more rapidly decreasing N fraction compared to Zn fraction with increasing O content are apparent from the data, it should be noted that there is natural scatter related to uncertainties in EDX measurements. While it is challenging to quantify the magnitude of this uncertainty, we note that repeated measurements at different sample locations lead to standard deviations of <1 at %. However, to provide an estimate for the uncertainty in composition, we measured a series of different samples with nominally identical oxygen contents of 2.1 at %. The standard deviations obtained from this sample set were used to estimate the uncertainty in composition, as represented by the error bars in Figure 1a. Figure 1b shows the Zn/N ratio as a function of the O content. For an O concentration lower than 10 at %, the Zn/N ratio is nearly constant. This constant Zn/N ratio hints at the formation of O interstitials (O_i_) or to incorporation of O on nitrogen vacancy sites (v_N_), resulting in substitutional oxygen states (O_N_) and reduced v_N_ concentrations. For O concentrations higher than approximately 10 at %, the Zn/N ratio increases, suggesting that O may replace N from its sublattice sites.

**Figure 2:**
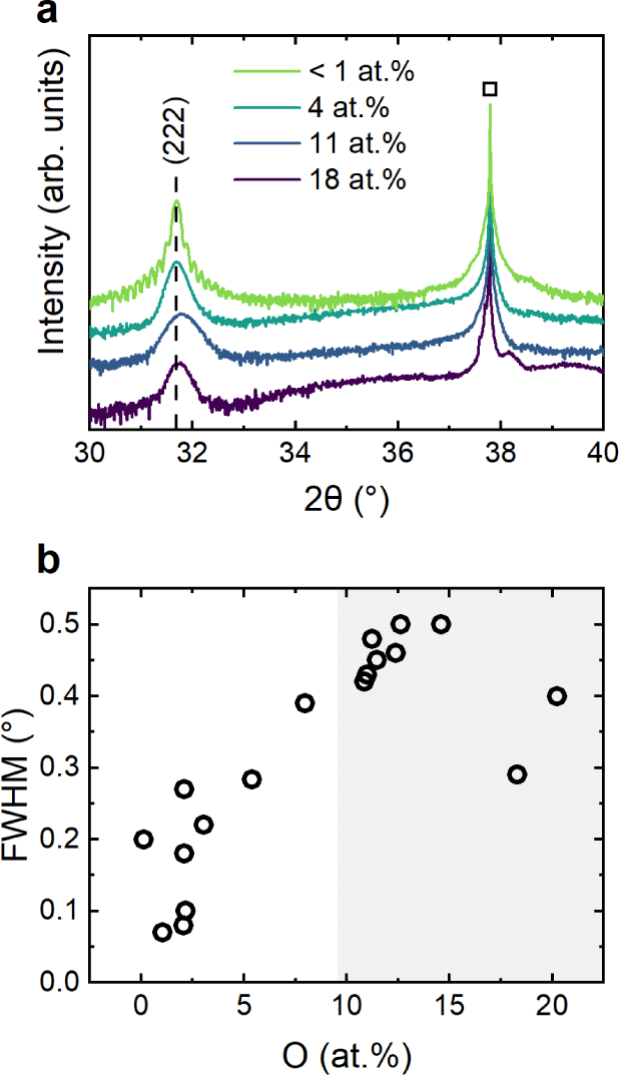
(a) Structural characterization of O:Zn_3_N_2_ as a function of O content. HR-XRD θ-2θ scan of (222)-oriented Zn_3_N_2_ thin films with different oxygen impurity contents, epitaxially grown on *a*-plane sapphire substrates (square). The legend indicates the experimentally determined oxygen content in each film. (b) fwhm of the (222) reflection determined by fitting of the data with a split Pseudo-Voigt function, and using a B-spline function for the background as a function of the O content. The gray background is a guide to the eye to emphasize the different regions discussed in the text.

Figure 2a shows representative high-resolution X-ray diffraction (HR-XRD) patterns of O:Zn_3_N_2_ films as a function of their oxygen concentrations. The reflections at 31.7° and 37.8° correspond to the (222) plane of Zn_3_N_2_ and the (11–20) plane of the *a*-plane Al_2_O_3_ substrate, respectively. The diffraction pattern is similar to the one reported by Oshima et al., who have reported the detailed growth mechanism and lattice alignment of (111)-oriented Zn_3_N_2_ on *a*-plane Al_2_O_3_.^9^ The pure film (<1 at % O) grown under optimized conditions shows a single (222) reflection from antibixbyite-type Zn_3_N_2_, with a full width at half maximum (fwhm) of 0.07°. With increasing oxygen content, the (222) reflection broadens and decreases in intensity. In particular, fitting of the HR-XRD patterns reveals that the fwhm increases from 0.07° to 0.50° as the O content increases from <1 at % to 15 at % (Figure 2b). However, for oxygen contents above 18 at %, the fwhm again decreases slightly, which may indicate a different O incorporation behavior in this range, as discussed below. Additional in-plane pole figure measurements of the {400} plane (Figure S1) show the 6-fold symmetric reflections that, as explained by Oshima et al.,^9^ arise due to twinning. While similar symmetry is observed for both the low oxygen-content and the oxygen-rich sample, the latter is characterized by broadening of the (040), (004) and (400) reflections and increased background signal, consistent with increased structural disorder and the possible presence of a minority amorphous phase. However, no other crystalline reflections are observed from any of the XRD measurements.

Within Zn_3_N_2_, the observed peak broadening likely arises from the incorporation of oxygen defects, such as O_N_ or O_i_, leading to structural defects. For example, this observation agrees with a study by Jiang et al.,^25^ who observed an increased XRD fwhm for sputtered bixbyite-type Ta_2_N_3_ films with increasing oxygen content. For oxygen contents >17 at %, we observe a decrease of the XRD fwhm but an increase of the scattering intensity between 34° and 37° that could arise from the presence of amorphous ZnON or ZnO regions. The (222) reflections shift slightly relative to pure Zn_3_N_2_, but the magnitude and direction do not shift monotonically with oxygen content. Thus, along with the impact of incorporated oxygen, we conclude that the shift is also affected by differences in lattice strain due to the uncertainty of the substrate temperature of ±10 °C during growth, as also reported in other studies.^8,24^ In addition, we observe an increase in the background signal between 33° and 37°, which could arise from increased crystalline disorder, or the presence of a minority amorphous ZnON or amorphous ZnO phase within or on the films.^26^ Nevertheless, we do not observe any indication for a crystalline ZnO phase or the (002) or (100) reflections from the metallic hexagonal Zn phase, which would be expected at 36.9° and 39.8°, respectively, based on reference mp-79 of the Materials Project.^27^ We note that the additional reflection at 38.2° corresponds to the Au (111) Bragg reflection, arising from Au contacts deposited on the top corners of the samples used for subsequent electrical transport measurements. Additionally, the influence of the film thickness on the fwhm from XRD is reported in Figure S2, indicating that differences in film thickness have a weak influence on the structural order compared to the oxygen content. Overall, we find that PA-MBE yields epitaxial (111)-oriented Zn_3_N_2_ thin films on *a*-plane sapphire, regardless of O content. However, the degree of impurity-induced disorder increases with increasing O content. The increased crystalline disorder with increasing O content is confirmed by AFM measurements performed on samples with different oxygen contents (Figure S3), showing a decrease in grain size with increasing oxygen content.

### 2.2 Electrical Properties of O:Zn_3_N_2_

**Figure 3:**
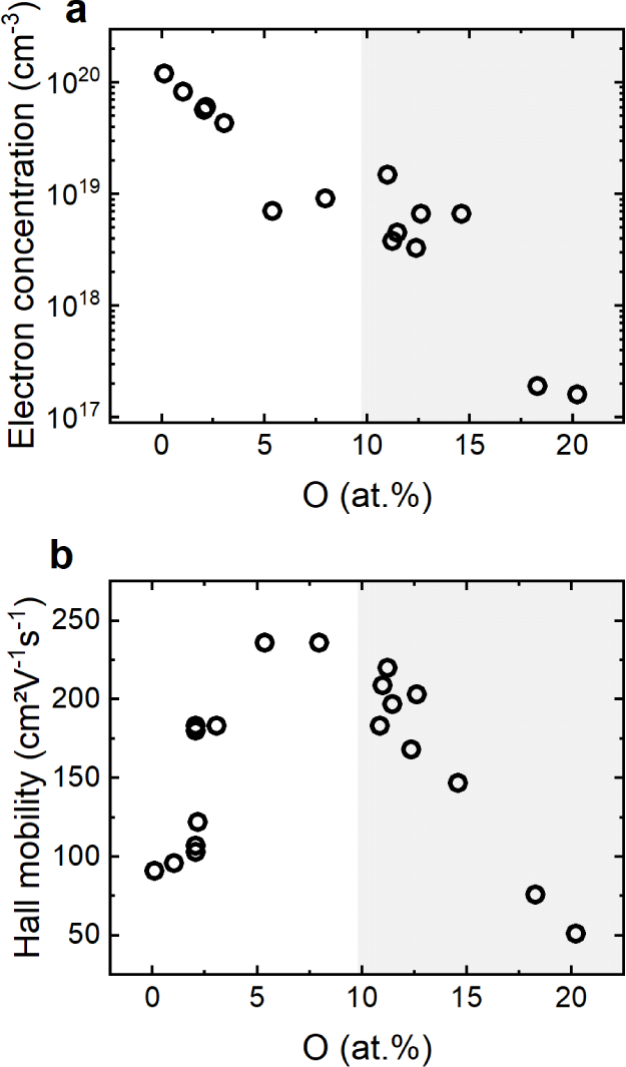
Electronic transport properties of Zn_3_N_2_ thin films as a function of their O content, obtained by room temperature Hall effect measurements. (a) Free electron concentration and (b) Hall mobility as a function of O content. The gray background is a guide to the eye to emphasize the different regions discussed in the text.

To understand the impact of increased O content on the electrical properties of O:Zn_3_N_2_ films, we first performed Hall effect measurements at room temperature. As expected, all films exhibited n-type conductivity, with the O content-dependent free electron concentration shown in Figure 3a. Consistent with previous reports on MBE-grown samples,^6,8^ we observe large electron concentrations between 2 × 10^19^ cm^–3^ and 2 × 10^20^ cm^–3^ for O contents lower than 5 at %, suggesting degenerate n^+^ conductivity (see below). However, for even larger oxygen contents of up to 20 at %, the charge carrier concentration decreases to 2 × 10^17^ cm^–3^. These values are lower than those typically reported for nonintentionally doped Zn_3_N_2_ but are similar to those that have been assigned to nondegenerate Zn_3_N_2_ thin films.^12,24^ This finding is surprising since substitutional oxygen impurities on nitrogen sites (O_N_) are typically implicated as being the origin of the large free carrier concentrations observed in Zn_3_N_2_.^22^ Considering that the degenerate character is a significant impediment to the development of Zn_3_N_2_ as a functional semiconductor, our observation of decreased electron concentration with increasing O content suggests an alternative source of this behavior and deserves careful attention, as discussed below.

In addition to the free carrier concentration, the Hall mobility also changes significantly with increasing oxygen content (Figure 3b). However, the dependence is not monotonic, and we can identify two distinct regimes. First, when oxygen content is increased up to 5 at %, we observe a strong increase in the mobility from 90 cm^2^ V^–1^ s^–1^ to 236 cm^2^ V^–1^ s^–1^. However, the mobility then decreases with increasing oxygen content, reaching a value of 50 cm^2^ V^–1^ s^–1^ at 20 at %. This decreasing mobility, which is especially pronounced for O contents >12 at %, is consistent with the increased fwhm of the (222) reflection of Zn_3_N_2_, speaking for a higher defect concentration in the material (Figure 2), which in large quantities also lead to an increased amount of crystalline disorder. For oxygen contents >17 at %, the mobility drops to 50 cm^2^ V^–1^ s^–1^, consistent with the observed increase of the scattering intensity between 34° and 37°, which likely arises from the presence of amorphous regions (Figure 2b).

**Figure 4:**
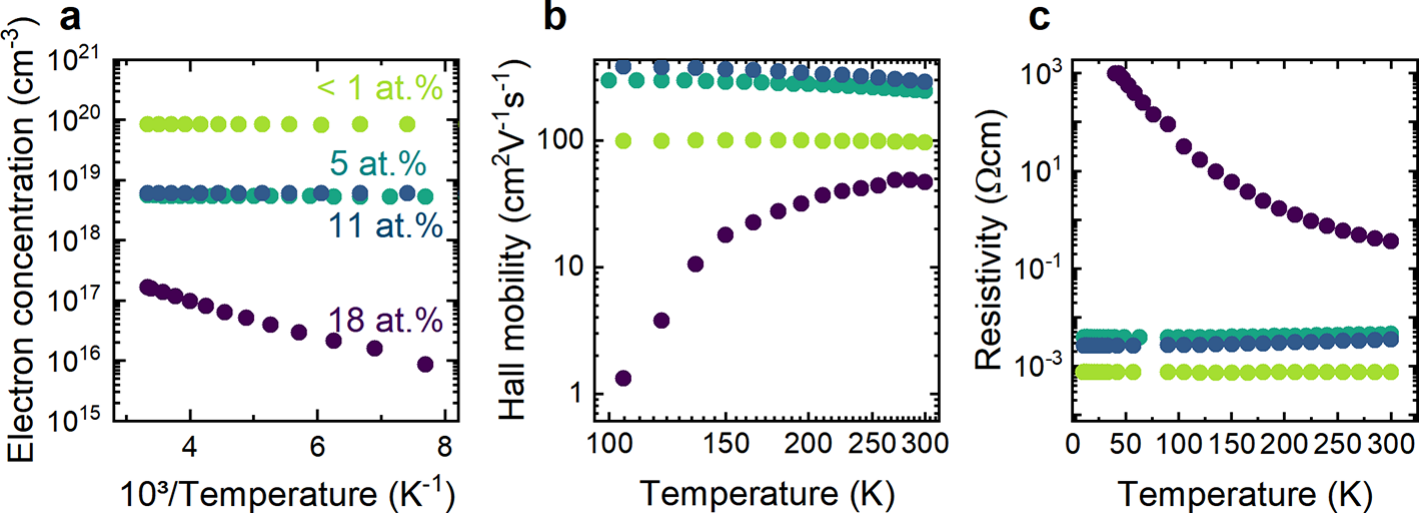
Temperature-dependent Hall effect measurements of four Zn_3_N_2_ samples with different oxygen concentrations. The samples with <1 at %, 5 at %, 11 at %, and 18 at % are represented in green, blue, cyan, and violet, respectively. (a) Free electron concentration as a function of inverse temperature. (b) Hall mobility and (c) resistivity as a function of temperature.

As a next step, we performed temperature-dependent Hall measurements on selected samples within these different regimes to evaluate the impact of oxygen impurities and structural defects on the transport characteristics of O:Zn_3_N_2_. Figure 4 shows the temperature-dependent charge carrier concentrations, Hall mobilities, and resistivities of O:Zn_3_N_2_ films with O contents of <1 at %, 5 at %, 11 at %, and 18 at %. For the three lowest oxygen content films, the charge carrier concentrations, mobilities, and resistivities remain approximately constant over the measured temperature range. Such behavior is indicative of the metallic conductivity of degenerate semiconductors that possess large free electron concentrations in the conduction band.^28^ In contrast, for 18 at % O, we observe a distinct temperature-dependent transport characteristic, with the charge carrier concentration decreasing from 1.7 × 10^17^ cm^–3^ to 8.6 × 10^15^ cm^–3^ when the temperature decreases from 300 to 90 K. Measurements performed at temperatures lower than 90 K resulted in unreliable results, most likely due to increased contact resistance. Fitting of the recorded temperature-dependence between 300 and 90 K with an Arrhenius function yields an activation energy of 30 meV, indicating a shallow donor state that is slightly smaller than the value of 80 meV reported for nondegenerate Zn_3_N_2_ grown by reactive sputtering at 100 °C.^12^ Because the complete freeze-out curve could not be collected, it is not possible to determine whether this represents the donor ionization energy or half the donor ionization energy. Nevertheless, we can conclude that the dominant donor state is a shallow level. The decreased charge carrier concentration with decreasing temperature, which is indicative of the semiconducting behavior of the higher oxygen content thin film, is also apparent from associated resistivity measurements. In particular, we observe an increased resistivity from 4 × 10^–1^ Ω cm to 1 × 10^3^ Ω cm with decreasing temperature from 300 to 50 K.

For this nondegenerate O:Zn_3_N_2_ film, the electron mobility increases with increasing temperature and reaches a maximum at 270 K. Fitting of this temperature dependence reveals that the mobility increases with a power of T^1^·^9^ between 150 and 210 K. However, for temperatures between 210 and 270 K, the temperature dependence is weaker, given by T^1^·^1^ and for temperatures above 270 K, the mobility slightly decreases. These results indicate that multiple scattering mechanisms contribute to the transport characteristics and have different impacts in different temperature regimes. In particular, ionized impurity-limited conduction, which is known to yield μ ∝ (*kT*)^1.5^,^29,30^ is likely dominant below 210 K. Between 210 and 270 K, scattering at dislocations (μ ∝ *kT*) may become more important, leading to the observed T^1.1^ dependence of the mobility. At even higher temperatures, the decreasing mobility is consistent with increasing contributions from phonon scattering, which has an ideal μ ∝ (*kT*)^−1.5^ dependence.^29,30^ However, the weaker temperature dependence observed in this regime suggests that both dislocation and phonon scattering contribute to the mobility. Overall, the composition- and temperature-dependent transport properties indicate that high oxygen concentrations enable the formation of nondegenerate Zn_3_N_2_ thin films. These nondegenerate films are characterized by shallow donor levels and moderate charge carrier mobilities that could be improved with reduced crystalline disorder.

### 2.3 Influence of Oxygen on Optical Properties of O:Zn_3_N_2_

**Figure 5:**
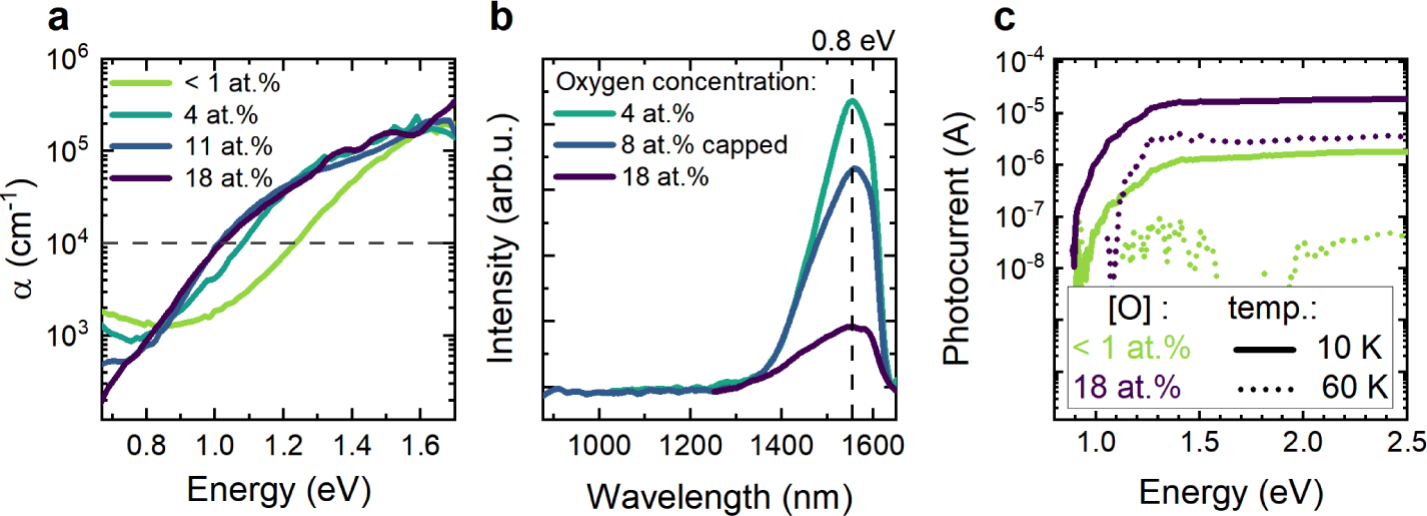
Optical properties of Zn_3_N_2_: (a) absorption coefficient α of Zn_3_N_2_ films with different oxygen concentrations as a function of photon energy, as determined by PDS. (b) Photoluminescence spectra collected at 4 K with 780 nm excitation on thin films with different oxygen contents. The sample with 8 at % O is capped with an 8 nm thick amorphous GaN protection layer (“capped”). (c) Photocurrent measurements performed on a pure Zn_3_N_2_ sample and a film containing 18 at % O at 10 K (solid line) and 60 K (short dots). The photocurrent was calculated by subtracting the dark current from the current measured under illumination. The dark currents measured at each temperature step are constant with temperature between 10 and 60 K for both samples and are in the 10^–4^ A range.

To better understand the influence of oxygen incorporation on the optoelectronic properties of Zn_3_N_2_ thin films, we next evaluated composition-dependent changes in the bandgap by means of photocurrent, absorption, and photoluminescence measurements, as shown in Figure 5. As a starting point, both band-edge and sub-bandgap optical absorption were quantified by photothermal deflection spectroscopy (PDS) for films with oxygen contents between <1 at % and 20 at % (Figure 5a). The optical absorption spectrum of the pure Zn_3_N_2_ thin film, with a charge carrier concentration of 1.2 × 10^20^ cm^–3^, shows strong sub-bandgap absorption, making it challenging to define a bandgap with the Tauc method.^31^ For this reason, we utilized the iso-absorption E_04_ method, which approximates the bandgap as the energy at which the absorption coefficient reaches 10^4^ cm^–1^.^32^ For the film with an O content <1 at %, this definition corresponds to a bandgap of 1.2 eV. With increasing oxygen contents from 4 at % to 14 at %, the bandgap decreases from 1.2 to 1.0 eV. For oxygen concentrations >14 at % and charge carrier concentrations <4 × 10^18^ cm^–3^, the bandgap remains unchanged at 1.0 eV (Figure 5a). This finding is consistent with the predictions from Kumagai et al.,^22^ who used density functional theory (DFT) to calculate the optical bandgap as a function of the charge carrier concentration, finding that it decreases by up to 0.8 eV when the charge carrier concentration in Zn_3_N_2_ decreases from 10^20^ to 10^18^ cm^–3^. From their calculations, the shift in optical bandgap is stronger when the charge carrier concentration is reduced from 10^20^ cm^–3^ to 10^19^ cm^–3^ than from 10^19^ cm^–3^ to 10^18^ cm^–3^. At a concentration of approximately 10^18^ cm^–3^, they find that the bandgap reaches its minimum value, suggesting the point at which the Mott transition to nondegenerate behavior occurs for n-type Zn_3_N_2_. This prediction is in excellent agreement with our experimental observations. Moreover, we observe decreasing sub-bandgap optical absorption with increasing oxygen content, which is consistent with the reduction of free carrier absorption in this spectral range according to the Drude theory.^33^ Based on these observations, we assign the decreasing bandgap with decreasing carrier concentration to the Burstein–Moss (BM) shift of degenerate semiconductors.^34,35^ At higher oxygen contents, and thus lower free electron concentration, we find that the optical bandgap of nondegenerate Zn_3_N_2_ is approximately 1.0 eV.

Since free carrier concentration and degeneracy influence the optical absorption spectrum, we next performed low-temperature photoluminescence (PL) measurements at 4 K to evaluate the emission properties as a function of oxygen content in Zn_3_N_2_ (Figure 5b). To investigate the impact of surface oxidation on the luminescence spectrum (see below), the sample containing 8 at % O was capped with an 8 nm thick MBE-grown amorphous GaN protection layer (denoted as “capped”) without breaking vacuum, which we have previously shown to protect Zn_3_N_2_ thin films against oxidation for periods of years.^36^ While all three films are characterized by an asymmetric emission peak located at 1520 nm (∼0.8 eV), the PL intensity decreases with increasing oxygen content, most likely due to enhanced nonradiative recombination. The emission energy agrees well with the onset of absorption in nondegenerately doped films observed in Figure 5a, as well as with the theoretically calculated bandgap between 0.86 eV, as modeled with HSE06, and 1.15 eV, as determined with G_0_W_0._^5^ Temperature-dependent PL measurements performed on the Zn_3_N_2_ layer with 4 at % O (Figure S5) show a decrease of the peak intensity and an increase of the fwhm with increasing temperature. The observed increase in the fwhm arises mainly from the increased spectral intensity at the high energy tail, which is typically observed in degenerate semiconductors due to an increased probability of transitions involving the recombination of free carriers.^37,38^ Power-dependent measurements performed on the same sample (inset Figure S5) show a linear dependence (slope of 1.1) of the PL peak intensity, which is consistent with direct band-to-band transitions.^39,40^ Here, we note that no PL signal was observed for the sample containing <1 at % O, which hints at rapid nonradiative recombination in this degenerately doped film. As discussed below, this finding is consistent with a high concentration of v_N_, which not only leads to large excess electron concentrations but also to a high density of nonradiative recombination centers.

To the best of our knowledge, photoluminescence from MBE-grown Zn_3_N_2_ samples has not been reported before. There are, however, reports on nanocrystals,^16,41^ hollow structures,^42^ microtips, nanowires,^43,44^ and sputtered Zn_3_N_2_ thin films.^37^ These studies show broadband emission between 0.85 and 0.90 eV, similar to the results obtained here.^16,41^ However, the groups studying Zn_3_N_2_ thin films deposited by reactive sputtering observed a narrow emission peak at 1.4 eV and a broader emission at 1.3 eV,^37^ which were attributed to band-to-band recombination and free carrier-related emission, respectively. After exposure to air, Trapalis et al.^20^ observed a low-energy broadband emission at 0.9 eV, which they attributed to in-gap states created by oxygen defects. However, they also reported an optical bandgap, determined via Tauc analysis, of 1.5 eV, which is considerably larger than our measured optical gap of 1.0 eV. In our case, no PL signal is observed at energies >1.1 eV. Furthermore, the PL emission from the capped Zn_3_N_2_ layer exhibits the same spectral contribution and shape as the uncapped film over the complete range of 0.75 to 1.4 eV. Together, the observations that the PL spectral shape is independent of the surface oxidation state, that the peak emission remains constant in energy but decreases in intensity with increasing oxygen content, and that the power dependence of the luminescence intensity displays a slope of approximately unity confirm that the measured PL signal arises from band-to-band recombination rather than from surface oxidation or oxygen-related defect centers.

To provide further insights into the optoelectronic characteristics of these films, we performed spectrally resolved photocurrent measurements at 10 and 60 K for both pure (<1 at % O) and doped (18 at % O) Zn_3_N_2_ (Figure 5c). While these layers possess similar charge carrier mobilities at room temperature, they have significantly different electron concentrations (Figure 3) that render them degenerate and nondegenerate, respectively. At 10 K, both films show a similar spectral response, with the photocurrent rapidly increasing with increasing photon energy and then saturating at excitation energies above 1.4 eV. However, the photocurrent is approximately 1 order of magnitude lower for the low oxygen content film. Furthermore, the onset of photocurrent is shifted to slightly lower energies with increasing oxygen content, as also observed in the room temperature optical absorption measurements due to the Burstein–Moss shift (Figure 5a). At 60 K, no photocurrent response is observed for the low oxygen content film (<1 at %), while a clear photoresponse is retained for the nondegenerate material (18 at % O). These observations suggest that carrier freeze-out at the lowest measurement temperature (4 K) enables a photoconductive response in both films. However, consistent with optical and transport measurements, thermal ionization of donor states leads to a large (metallic) dark current and suppression of the photoconductivity for the <1 at % film at higher temperatures.

## 3 Discussion

As described above, we find that the oxygen content in Zn_3_N_2_ films plays a critical role in defining their optoelectronic properties, especially with respect to the transport characteristics that have thus far limited their utility as functional semiconductor thin films. Surprisingly, our results indicate that low oxygen contents promote the formation of O_N_ and/or v_N_, which result in degenerate n-type conductivity and a pronounced Burstein–Moss shift, while high oxygen contents lead to nondegenerate, semiconducting films with the expected bandgap near 1.0 eV. This finding cannot be described by a simple model of substitutional oxygen incorporation on nitrogen sites (O_N_), since such states should act as donors, increasing the free electron concentration. Thus, it is important to also consider the role of native point defects alongside these oxygen impurities in Zn_3_N_2_. Although experimental studies are limited, DFT-based theoretical predictions by Kumagai et al.^22^ indicate that the lowest formation energy point defects in Zn_3_N_2_ are nitrogen vacancies (v_N_), which are triple donors, and substitutional oxygen impurities (O_N_), which are single donors. However, when the chemical potential of N is increased, as in the case of materials grown here by PA-MBE with an atomic nitrogen source, other native defects must also be considered. These defects include zinc vacancies (v_Zn_), which are double acceptors, and zinc interstitials (Zn_i_), which are double donors, as well as hydrogen impurities (H_i_) and nitrogen interstitials (N_i_). In Zn_3_N_2_, such N_i_ defects are particularly complex and can comprise a diatomic molecule in various structural and electronic configurations, resulting in possible charge states of 1–, 1+, 2+, or 3+. Thus, such defects can serve either as donors or acceptors with differing manifolds of electronic states within the bandgap.^22^

These considerations regarding the nature and formation energies of both native and impurity defects enable a qualitative interpretation of the present results, as well as insights into previously reported findings and a basis for rationally controlling the optoelectronic properties of Zn_3_N_2_ films. In particular, while O_N_ can contribute to the degenerate character of unintentionally doped films (<1 at % O), our findings indicate that the presence of v_N_, which serve as triple donors and are characterized by low formation energies, also play a critical role in this behavior. Importantly, this assignment enables a revised interpretation of the previously reported nondegenerate character of Zn_3_N_2_ films formed via reactive sputtering at low substrate temperature.^12^ In that work, the authors concluded that the reduced substrate temperature is essential for maintaining moderate carrier concentrations, which they attributed to suppressed O_N_ formation. However, it is also important to recognize that the concentration of v_N_, which are thermodynamically defined defects, should decrease exponentially with decreasing substrate temperature. MBE-grown Zn_3_N_2_ samples have been reported to have higher charge carrier concentrations than samples grown with other deposition techniques.^11,20,45^ Despite the exceedingly high purity that can be achieved by MBE, large concentrations of v_N_, each donating up to three electrons, can be generated at the low pressures associated with this method.

In the present work, when oxygen is introduced into the growth chamber, O occupies a fraction of the otherwise vacant nitrogen sites as an O_N_ defect. This substitution partially passivates the v_N_ sites, leading to the donation of one rather than three electrons. Not only does this hypothesis explain the observed decrease in charge carrier concentration, but also the increase in mobility, when the oxygen content increases from <1 at % to 5 at % (Figure 4). In particular, Park et al.^46^ used DFT to study the influence of O atoms on v_N_ sites in a Zn_3_N_2_ lattice, concluding that v_N_ sites lead to reduced mobilities, whereas oxygen passivation in the form of O_N_ results in enhanced mobilities. At sufficiently high temperatures, such a conclusion is consistent with the higher charge state of triply ionized v_N_ compared to singly ionized O_N_. While this finding is consistent with our observations for oxygen contents up to 5 at %, we also observe a decrease of the mobility as the oxygen concentration increases from 9 at % to 20 at %. This decreased mobility likely arises from increased impurity scattering and crystalline disorder in the material, as indicated by the comparatively large fwhm in the HR-XRD patterns over this range of oxygen concentrations (Figure 2).

Our finding that the dominant donor in nondegenerate Zn_3_N_2_ is shallow (i.e., statistically thermally ionized at room temperature) suggests that oxygen passivation of v_N_ can only account for a decrease of the free electron concentration by a factor of approximately three. Thus, additional mechanisms for decreasing the carrier concentration by several orders of magnitude at higher oxygen contents must also be considered. While prior studies have invoked bandgap opening as being key for enabling nondegenerate character, we find no increase of the bandgap with decreasing charge carrier concentration; rather, we observe a decrease in the optical bandgap as the Burstein–Moss shift becomes less pronounced (Figures 3 and 5). Thus, we conclude that additional compensating acceptor states must be generated, thereby further reducing the free electron concentration. Here, PA-MBE growth under nonequilibrium conditions, which are defined by the high chemical potential of atomic nitrogen, can have a significant influence on defect formation energies, opening up a broader range of potential compensating states.

We consider three different defect types that are likely to be generated, with different probabilities for different oxygen concentrations. First, we note that the antibixbyite lattice possesses structural vacancy sites that can potentially host large concentrations of interstitial oxygen impurities (O_i_), which are expected to serve as double acceptors. Such a charge compensation mechanism has been previously observed in bixbyite-type Ta_2_N_3_, leading to a lifting of its degenerate conductivity.^25^ Indeed, the composition analysis presented in Figure 1 reveals that, for oxygen contents <10 at %, the Zn/N ratio remains approximately constant with increasing O content, suggesting the formation of O_i_, which could be favored by the presence of these antibixbyite structural vacancy sites. Second, for oxygen contents ≥10 at %, an increase of the Zn/N ratio with increasing O content is observed, suggesting the formation of O_N_, though we cannot eliminate the possibility for simultaneous formation of lower concentrations of compensating v_Zn_. Third, for high oxygen contents, the high defect density may favor the formation of N_i_. Figure S6 shows fluorescence-yield X-ray absorption spectroscopy data (XAS) performed at the N K-edge for three O:Zn_3_N_2_ thin films with oxygen concentrations of <1 at %, 11 at %, and 18 at %. The absorption spectra of all three samples are similar except for an additional peak at 401.1 eV observed for the nondegenerate sample (18 at % of O). A similar spectral contribution was previously reported in other nitride compounds to stem from intercalated molecular nitrogen N_2_.^47−49^ Thus, this feature could arise from intercalated N_2_ or from defects involving N_i_ and leading to N–N distances close to N_2_, highlighting the possible formation of additional compensating defect states at higher oxygen contents.

## 4 Conclusion

In summary, MBE-grown samples help elucidate the fundamental properties of Zn_3_N_2_ and the role of oxygen in the growth of nondegenerate zinc nitride. First, HR-XRD and AFM measurements show a change in crystal quality with increasing O contents. Second, the optical bandgap determined by photocurrent and PL measurements does not change with the oxygen concentration and is constant at 1.0 and 0.8 eV, respectively. However, with photothermal deflection spectroscopy, the optical bandgap energy decreases from 1.2 to 1.0 eV for O contents <11 at %, due to the Burstein–Moss shift, but remains constant at 1.0 eV for higher oxygen contents. By increasing the oxygen content, it was possible to tune the charge carrier concentration from 10^20^ cm^–3^ to 10^17^ cm^–3^ and to create nondegenerate semiconductors suitable for optoelectronic applications, such as thin-film solar cells or transistors. This decrease in charge carrier concentration is likely achieved by off-stoichiometry, leading to the formation of acceptor defects such as O_i_ that influence the doping level in the material. For high oxygen contents >17 at %, the high defect density likely leads to the formation of additional defects, such as N_i_, as supported by XAS measurements. Overall, our work shows that controlled oxygen incorporation is beneficial for obtaining nondegenerate zinc nitride. This strategy could be applied to other degenerate, nominally semiconducting nitrides, potentially allowing other previously abandoned binary compounds to be again attractive for technological applications.

## 5 Experimental Section

### 5.1 Synthesis of Zn_3_N_2_

Thin film Zn_3_N_2_ samples with variable oxygen impurity content were grown with a Riber 32 molecular beam epitaxy (MBE) system equipped with a Zn effusion cell and a radio frequency plasma source. High-purity Ar, O_2_, and N_2_ (7.0 Ar, 7.0 N_2_, and 6.0 O_2_, Linde) gases were supplied to the plasma source, and the mixing ratio was controlled with mass flow controllers. Ar was used to ignite the plasma for high-purity Zn_3_N_2_ growth and to avoid the introduction of O_2_ into the chamber. For deposition of O-doped samples, different doses of O_2_ were added to the main chamber before growth. Prior to growth, the substrate was mechanically and ultrasonically cleaned for 5 min in acetone and 5 min in isopropanol, after which it was dried with flowing nitrogen. The substrate was then immediately loaded onto the sample holder and stored in the load lock of the MBE chamber. Following transfer to the growth chamber, the surface was conditioned via exposure to N_2_ plasma for 10 min. Growth was then performed at a set point substrate temperature of 150 °C, with typical uncertainties of ±10 °C, on single-side polished *a*-plane sapphire for all samples.

### 5.2 Structural Characterization

High-resolution X-ray diffraction (HR-XRD) measurements were performed with a Rigaku SmartLab 3 kW system equipped with a Cu anode X-ray source. A Ge(220) × 2 monochromator was placed in the incident beam path to selectively provide Cu–Kα_1_ radiation. After sample surface alignment, the substrate (11–20) reflection was used for precise alignment of each sample to enable comparison of the HR-XRD patterns from the different samples. The θ–2θ scans were performed from 30° to 40° with 0.01° steps and a scan speed of 0.5°/min. Film morphologies were determined using a Veeco MultiMode atomic force microscope (AFM) in tapping mode with NSG30 tips from TipsNano (tip height 14–16 μm, tip radius of curvature:10 nm).

### 5.3 Elemental Characterization

Energy dispersive X-ray spectroscopy (EDX) was performed using a Bruker XFlash6130 energy-dispersive X-ray detector with an analysis spot size of 1 μm. The detector was mounted on a Zeiss EVO MA10 scanning electron microscope (SEM) operated with an electron beam energy of 5 keV to limit the penetration depth and suppress the substrate signal. The resulting EDX spectra were quantified using the Phi(rho,z) method and deconvolution function SeriesFit. Since EDX is often subject to quantitative uncertainties, the compositions of four selected samples were also characterized by elastic recoil detection analysis (ERDA) and Rutherford backscattering spectrometry (RBS), which enabled depth-dependent elemental quantification. ERDA and RBS were performed at Helmholtz-Zentrum Dresden-Rossendorf (HZDR) using a 43 MeV Cl^7+^ ion beam. The angle between the sample normal and the incoming beam was 75°, and the scattering angle was 30°. The analyzed area was ∼2 × 2 mm^2^. The recoil atoms and scattered ions were detected with a Bragg ionization chamber, which enables energy measurement and identification of the atomic number, Z, of the elements within the film. ERDA and RBS spectra were fitted simultaneously with the program Windf v9.3g.^50^

As summarized in Table 1, the oxygen concentrations of samples grown without the intentional introduction of oxygen were determined to be 4 at % when measured via EDX but only 0.6 at % as quantified by ERDA. Similarly, the sample containing 20 at % oxygen according to EDX, yielded a concentration of 17 at % oxygen by ERDA. The 3 at % lower oxygen content measured by ERDA compared to EDX could be due to contributions from the substrate, which contains oxygen, to the EDX yield or from the nature of EDX, which is known to overestimate light element concentrations.^51^ However, the trend measured with EDX is confirmed by ERDA, which is known to be more accurate for lighter elements and allows for the selection of the depth region for the calculation of the oxygen concentration given its depth sensitivity. For this reason, we recalibrated the oxygen content measured by EDX using the ERDA results by subtracting the excess 3 at % oxygen content measured by EDX and then renormalizing the sum of the elemental contributions from Zn, O, and N to 100 at %.

**Table 1:** Zn, O, and N Content for Four Samples Determined with ERDA/RBS and EDX Measurements

	ERDA			EDX		
Samples	Zn (at %)	O (at %)	N (at %)	Zn (at %)	O (at %)	N (at %)
**1**	56.8	16.8	26	48	21	31
**2**	60	0.58	39.2	55	4	41
**3**	58.7	13.9	27.1	53	17	30
**4**	57.4	11.6	30.9	47	15	38

### 5.4 Optoelectronic Characterization

Optical absorption characteristics were determined with a home-built photothermal deflection spectroscopy (PDS) setup, with the sample placed into a cuvette filled with perfluorohexane. The excitation beam provided by the monochromatized light of a xenon lamp (Short Arc XBO 150 W, OSRAM) was directed to the sample at normal incidence, while a red probe laser diode (CPS635, Thorlabs) was aligned parallel to the surface of the sample. The deflected probe laser beam was detected by a 2-segment photodiode. Lock-in amplification (5210, Princeton Applied Research) of the signal was performed via chopping of the optical excitation beam at 9 Hz. For quantification of the absorption coefficient from the measured PDS signal, the sample thickness was determined with a stylus profilometer.

Photocurrent measurements were performed with a home-built setup. The sample was connected to a Keithley 2400 source meter with top contacts (20 nm Ti as adhesion layer and 80 nm Au) and placed into a contact gas He-flow cryostat (Model Optistat CF2, Oxford Instruments). The temperature was controlled from 4 to 300 K with a Mercury iTC temperature controller (Oxford Instruments). The sample was illuminated with monochromatic light provided by a xenon lamp (Short Arc XBO 450 W, OSRAM) coupled to a Jobin Yvon TRIAX 550 monochromator with 1200/500 gratings. The covered spectral range was from 1400 to 300 nm, and the step size was 5 nm. The resulting photocurrent was measured under an applied bias of 0.01 V after allowing the temperature to stabilize for 30 min and was calculated as the difference between the current under monochromatic illumination and the dark current.

Room-temperature Hall measurements were conducted with an EGK Hem-2000 Hall effect measurement system with an applied magnetic field of 0.58 T. Temperature-dependent Hall measurements were performed using a closed cycle cryostat integrated into a LakeShore Model 8404 AC/DC Hall Effect Measurement System operated in DC mode with a magnetic field of 0.9 T. For all measurements, electrical contacts comprising 20 nm Ti/80 nm Au stacks with the van der Pauw geometry were deposited through a shadow mask via e-beam evaporation (PRO Line PVD 75, Kurt J. Lesker Company).

Photoluminescence (PL) measurements were conducted with a home-built confocal microscopy setup equipped with an apochromatic objective with a numerical aperture of 0.81. The samples were mounted in a closed-cycle cryostat operating in the 4–300 K temperature range and were excited with a 780 nm continuous wave laser with a power of 2.5 mW (790 W cm^–2^). The collected signal was sent to a spectrometer with a focal distance of 750 mm (Andor Shamrock 750, grating SR5-GRT-0150 and SR5-GRT-1250) and was analyzed using a CCD (Andor iDus InGaAs detector, 1024 pixel). The power density for the power-dependent measurements was changed from 158 W cm^–2^ to 885 W cm^–2^.

XAS measurements at the N K-edge were carried out at the IOM-CNR BEAR beamline at Elettra. All samples were measured in fluorescence mode with the vertical slit set to 50 μm. Three energetic regions of the N K-edge were defined for measurement, scanned as follows: (i) 390 to 402 eV with a step size of 0.25 eV, (ii) 402 to 423 eV with a step size of 0.05 eV, and (iii) 423 to 460 eV with a step size of 0.2 eV. Each scan was repeated twice, the integration time per point was set to 0.4 s, and the incident X-ray intensity was measured before and after analysis of the samples. The Athena software was used to remove pre- and postedge contributions and to normalize the data to the edge step. Each spectrum was energy-calibrated using the second derivative of the corresponding reference absorption spectrum.

## Data Availability

The data that support the findings of this study are available from the corresponding author upon reasonable request.

## Supporting Information Available

AFM images, power-dependent and temperature-dependent PL measurements, and EXAFS spectra for samples with different oxygen contents (PDF)

## Abbreviation

O:Zn_3_N_2_: oxygen-doped Zn_3_N_2_
