# Supplementary material for: Oxygen Incorporation as a Route to Nondegenerate Zinc Nitride Semiconductor Thin Films
Source: ACS Appl Mater Interfaces. 2025 Jan 28;17(5):7958–68. doi: 10.1021/acsami.4c16921 (PMC11803553; doi:10.1021/acsami.4c16921)
Supplement: Supplementary file 1 — am4c16921_si_001.pdf [file am4c16921_si_001.pdf]

Supporting information for:

Oxygen incorporation as a route to non-degenerate zinc nitride semiconductor thin films

Elise Sirotti,[1,2] *Bianca Scaparra,*[3] *Stefan Böhm,*[1,2] *Florian Pantle,*[1,2] *Laura I. Wagner,*[1,2] *Felix Rauh,*[1,2] *Frans Munnik,*[4] *Chang-Ming Jiang,*[1,2] *Matthias Kuhl,*[2] *Kai Müller,*[3] *Johanna Eichhorn,*[2] *Verena Streibel,*[1,2] *Ian D. Sharp* [1,2]*

[1] Walter Schottky Institute, Technical University of Munich, 85748 Garching, Germany
[2] Physics Department, TUM School of Natural Sciences, Technical University of Munich, 85748 Garching, Germany
[3]TUM School of Computation, Information and Technology, and MCQST, Technical University of Munich, 85748 Garching, Germany
[4] Helmholtz-Zentrum Dresden-Rossendorf, 01328 Dresden, Germany

* sharp@wsi.tum.de

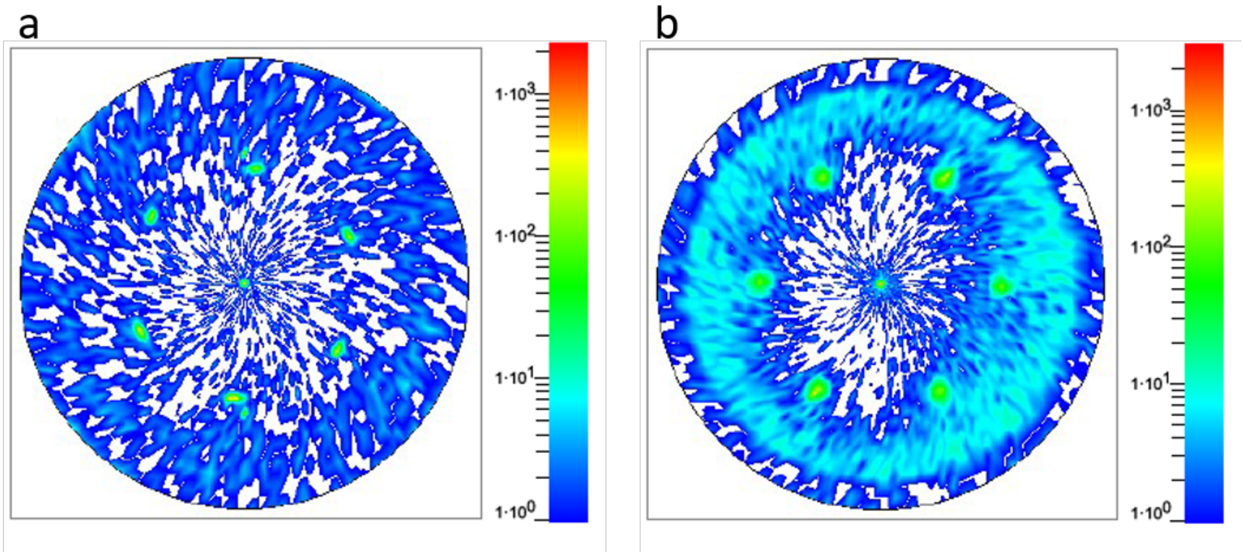

Figure S1. In-plane pole figure measurement of the Zn_3N_2 {400} diffraction peaks, showing the 6-fold symmetric reflections due to the twinning of the lattice plane, as explained by Oshima et al.[1] a) For Zn_3N_2 with 5 at.% of O, b) for Zn_3N_2 with 24 at.% of O.

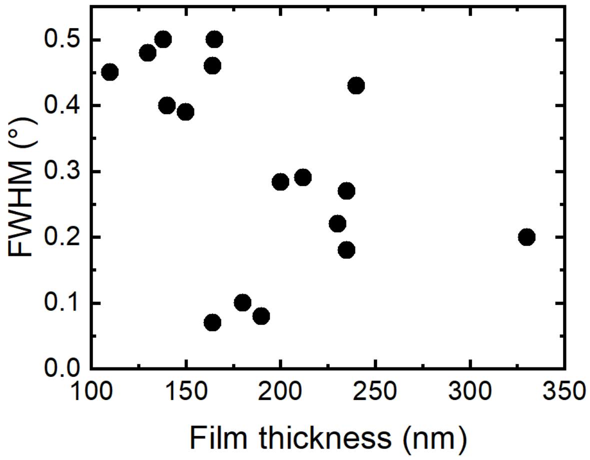

Figure S2. XRD full-width at half maximum (FWHM) as a function of the film thickness.

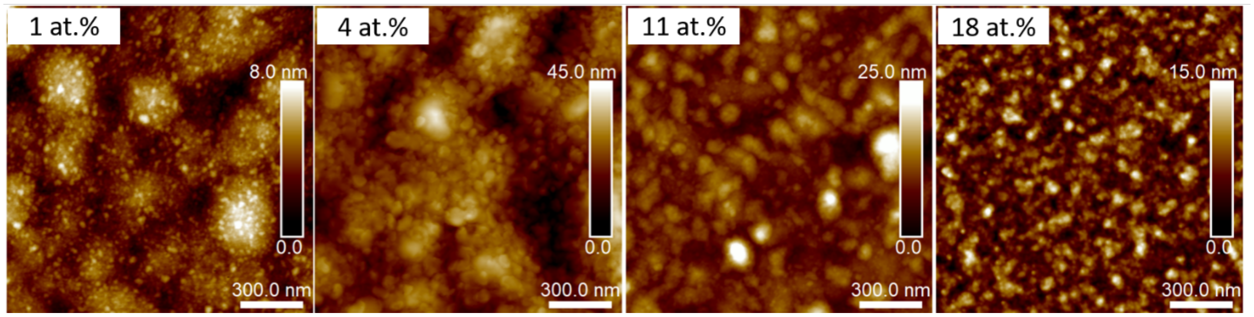

Figure S3. Atomic force microscopy measurements on Zn_3N_2 films with different oxygen concentrations, as indicated in the top left of each image. The small grains observed on top of the samples, especially visible on the sample with 1 at.% of oxygen (left), are not observed on top of samples protected with a GaN capping layer (Figure S4), which led to the conclusion that the small grains are a consequence of surface oxidation.

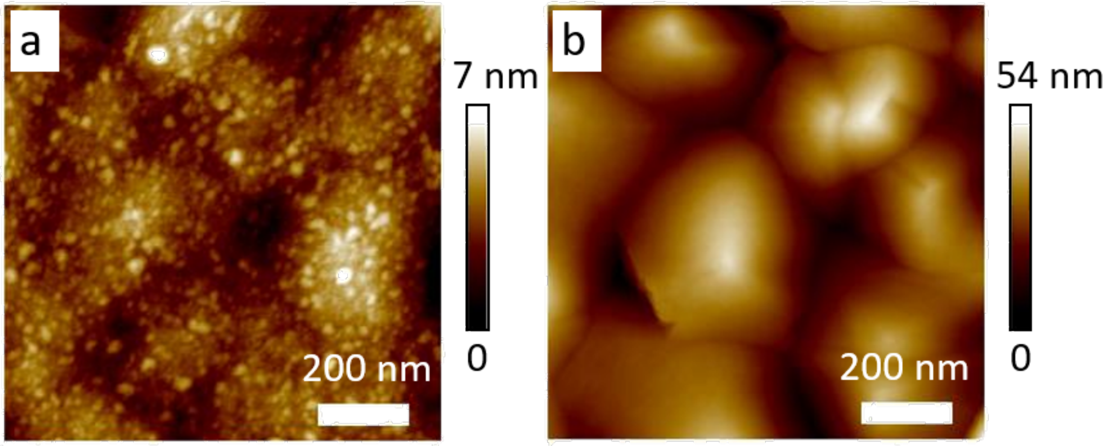

Figure S4. AFM images for an uncapped sample (a) and a sample capped with an 8 nm thick GaN capping (b),[2] both with an oxygen concentration < 1 at.%.

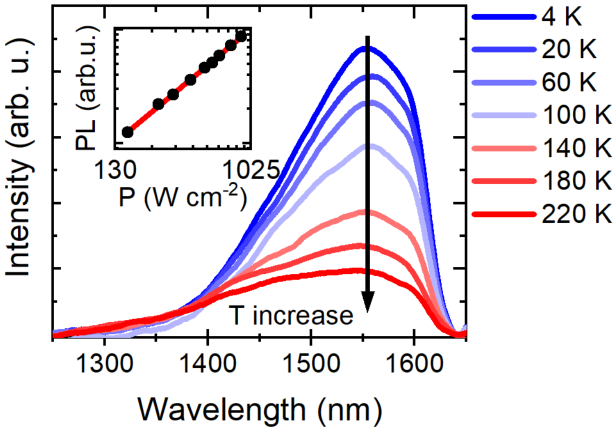

Figure S5. Photoluminescence of a Zn_3N_2 layer with 4 at.% of oxygen. Temperature-dependent measurements show a decrease in the PL intensity with temperature. (inset) Maximum of the PL signal as a function of the laser power density, with a linear fit showing a slope of 1.1.

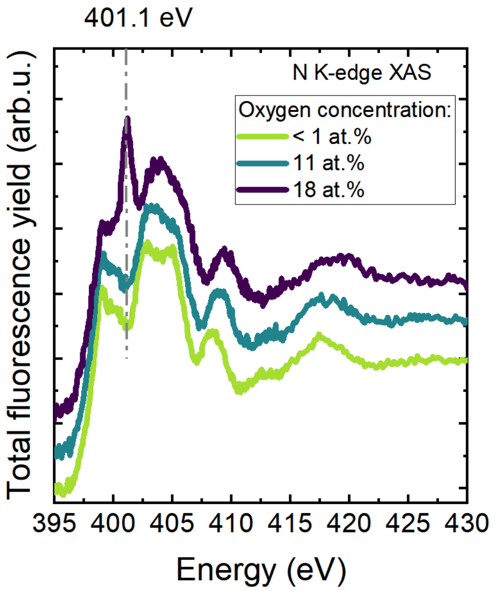

Figure S6. Fluorescence-yield XAS measurements were performed on the N K-edge for three samples with different oxygen concentrations. For the sample with 18 at.% oxygen, an additional peak at 401.1 eV appears, often assigned to interstitial molecular nitrogen.

References

1. Oshima, T. & Fujita, S. (111)-Oriented Zn 3 N 2 Growth on a-Plane Sapphire Substrates by Molecular Beam Epitaxy. *Jpn. J. Appl. Phys.* **45,** 8653; 10.1143/JJAP.45.8653 (2006).

2. Sirotti, E., Böhm, S. & Sharp, I. D. Ultrastable Zn 3 N 2 Thin Films via Integration of Amorphous GaN Protection Layers. *Adv Materials Inter* **11**; 10.1002/admi.202400214 (2024).
